# Community pharmacists’ acceptability of pharmacist-delivered depression screening for older adults: a qualitative study

**DOI:** 10.1007/s11096-023-01581-1

**Published:** 2023-04-20

**Authors:** Duha N. Gide, Sarira El-Den, Yee Lam Elim Lee, Natasa Gisev, Kevin Ou, Claire L. O’Reilly

**Affiliations:** 1 Sydney Pharmacy School, Faculty of Medicine and Health, The University of Sydney, Building Number A15, Science Rd Camperdown, Sydney, NSW 2006 Australia; 2 National Drug and Alcohol Research Centre, UNSW Sydney, Sydney, NSW Australia; 3 Pharmaceutical Society of Australia, Sydney, NSW Australia

**Keywords:** Community pharmacist, Depression, Older adult, Screening

## Abstract

**Background:**

Late-life depression often goes underdiagnosed and undertreated, affecting the quality of life of older adults. Pharmacists are well-placed to identify older adults who may be at risk of depression by using appropriate screening tools.

**Aim:**

To explore community pharmacists’ acceptability of performing late-life depression screening in Australian community pharmacies.

**Method:**

Semi-structured interviews with community pharmacists were conducted to gauge their perceptions regarding delivering depression screening services for older adults. Data analysis was conducted using an iterative, inductive approach. Key themes were identified, which were further explored and divided into subthemes. Subthemes were categorised as either barriers or facilitators. Each subtheme was mapped to the Capability, Opportunity, Motivation-Behaviour model by classifying whether they impacted pharmacists’ capability, opportunity, or motivation regarding depression screening.

**Results:**

Fifteen pharmacists were interviewed, 12 of whom were female and 11 of whom practised in a metropolitan area. Four key themes were identified including: training needs, environmental factors, pharmacists’ roles, and organisational support, which were further divided into 13 subthemes. Three subthemes were mapped to Capability, seven to Opportunity and three to Motivation. Barriers included lack of resources and lack of remuneration, while facilitators included training, pharmacists’ accessibility, and rapport with consumers.

**Conclusion:**

The findings of this study demonstrate that while community pharmacists found depression screening for older adults in community pharmacies to be an acceptable service, there remains a need for the development of funding schemes and standardised guidelines for pharmacist-delivered depression screening for older adults.

**Supplementary Information:**

The online version contains supplementary material available at 10.1007/s11096-023-01581-1.

## Impact statements

- Community pharmacists may utilise their accessibility and rapport with consumers to facilitate the implementation of late-life depression screening services in community pharmacies, thereby contributing to early detection and treatment of late-life depression.
- Standardised guidelines and remuneration pathways may be developed to support pharmacists in delivering late-life depression screening.

## Introduction

Depression affects approximately 5% of the world’s adult population [1], while late-life depression (LLD) affects between 10 and 40% of community-dwelling older adults (aged 65 years and older) [2, 3]. LLD can negatively impact the quality of life of older adults, resulting in significant distress and functional and cognitive impairment [2]. LLD has also been linked to increased rates of suicide and non-suicide mortality [4]. Therefore, it is imperative that LLD be diagnosed and treated in a timely manner, so as to prevent premature mortality and improve the overall quality of life of older adults with depression [2].

Depression in older adults often goes undiagnosed and untreated [5]. Multiple factors are attributed to this, including a lack of awareness pertaining to the difference in the presentation of depression in older adults compared to younger adults [6]. Symptoms such as fatigue, insomnia and a loss of interest in living commonly affect older adults with depression, while younger adults are more likely to experience dysphoria and feelings of worthlessness [6]. Older adults also often associate symptoms of depression as a normal consequence of ageing and fail to report these symptoms to healthcare professionals (HCPs) [7]. Hence, a lack of education and awareness surrounding symptoms of LLD may result in failure to seek treatment [8].

Pharmacists are one of the most accessible and frequented HCPs [9]. Consumers are up to 10 times more likely to visit a community pharmacy compared to a general practitioner (GP) [9], and on average, Australians visit community pharmacies 18 times per year [10]. Their accessibility and frequent interactions with consumers allow them to recognise changes in mood or behaviour indicative of mental illness, thereby ideally placing them to identify, screen, and refer older adults at risk of depression [11], which is important as 1 in 7 Australians have experienced depression in their lifetime [12]. Community pharmacists have also demonstrated an increasing role in the early prevention and treatment of a range of illnesses/risk factors, including cardiovascular disease and osteoporosis [13]. Additionally, consumers view community pharmacies as a “safe space” to discuss mental health concerns [14, 15]. A systematic review by Davis et al. identified nine studies which demonstrated that collaborative teams of HCPs, including pharmacists, improved mental health clinical outcomes [16]. Furthermore, a systematic review by Miller et al., identified 10 studies which explored the feasibility of pharmacist-delivered depression screening in adults aged 18 years and older [17]. Seven studies demonstrated that pharmacists could effectively recognise consumers with undiagnosed depression using screening tools, highlighting the potential role of pharmacists in depression screening. However, there remains an unmet need for the early identification of LLD.

Acceptability refers to the perception that a certain service or practice is satisfactory to stakeholders, and is a major determinant affecting the design, evaluation, and provision of healthcare services [18]. As such, pharmacists’ acceptability of screening services should be assessed. A systematic review conducted by El-Den et al. investigated key stakeholders’ (i.e., pharmacists, consumers, and other HCPs) acceptability of community pharmacist-delivered screening [13]. They found that pharmacist-delivered screening for various medical conditions/risk factors was acceptable to key stakeholders. Therefore, while there are studies that have explored pharmacists’ acceptability regarding screening for various medical conditions, such as atrial fibrillation [19, 20] and hypertension [21], there is a lack of data pertaining to the acceptability of pharmacist-delivered LLD screening from the perspectives of community pharmacists.

### Aim

This study aimed to explore community pharmacists’ acceptability of delivering LLD screening services. The research focused on how pharmacists’ capability, opportunity and motivation may influence the provision of pharmacist-delivered LLD screening services and sought to identify barriers and facilitators which may impact the implementation of such services.

### Ethics approval

Ethics approval was obtained from The University of Sydney Human Research Ethics Committee [2020/074] on 3 March 2020.

## Method

### Design

A qualitative study consisting of semi-structured interviews with community pharmacists was used to explore their perceptions of the acceptability of pharmacist-delivered depression screening for older adults aged over 65 in community pharmacy settings.

### Development of study instrument

The research team developed an interview guide based on previous research on the acceptability of perinatal depression screening [22], the feasibility of depression screening [23], and the acceptability of community pharmacist-delivered screening [13] (Supplementary material 1). Interview questions explored pharmacists’ views on the early intervention for depression, the use of depression screening tools, and barriers and facilitators affecting pharmacist-delivered LLD screening. Several interviews with community pharmacists were conducted by EL to pre-test the interview guide and ensure face validity.

### Study procedures

Participants were considered eligible for inclusion if they were currently practising as a community pharmacist in Australia. Pharmacists were recruited by disseminating promotional emails through the Pharmaceutical Society of Australia, and social media platforms, including Twitter and Facebook, and through word-of-mouth. Pharmacists were informed that interviews would be conducted via telephone or video conferencing software (i.e., Zoom) by a pharmacy student researcher (EL). Pharmacists provided written consent to participate in the study via email prior to the interview and consent for audio-recording during the interview. Interviews were conducted between March and October 2020 until data saturation was reached.

### Data analysis

Interviews were audio-recorded and transcribed verbatim. All transcripts were checked initially by EL, and then by DG to ensure the accuracy of the interview transcripts. Transcripts were then imported into NVivo 12 software [24] and de-identified and coded by DG, a Mental Health First Aid (MHFA)-trained community pharmacist with experience in pharmacy practice and educating pharmacy students. Thematic analysis was used to identify themes using an iterative, inductive approach. Each theme was explored in detail and further divided into subthemes. Each subtheme was categorised as either a barrier or facilitator and then mapped to the corresponding element of the Capability, Opportunity, Motivation-Behaviour (COM-B) model [25].

The COM-B model is a useful framework to identify the factors which may be enforced or adapted to encourage the development and implementation of an intervention [25]. According to the COM-B model, three conditions are needed to elicit a behaviour change: capability, opportunity, and motivation. Capability encompasses an individual’s psychological and physical capacity to partake in an intervention. Opportunity considers the external factors that prompt a certain behaviour [25], either provided by environmental systems (physical opportunity), or involving other people (social opportunity) [26]. Meanwhile, motivation represents the mental processes that direct behaviour and can take form as automatic motivation (including instinctive processes) or reflective motivation (which involves conscious thought processes) [26]. The COM-B model has previously been used to provide insight into factors affecting various healthcare service implementations, including weight management services in community pharmacies [27]. DG conducted initial mapping and development of coding framework, and regular meetings were held by COR, SE, and DG to refine and agree on themes and mapping until consensus was reached.

## Results

A total of 15 pharmacists participated in semi-structured interviews. Six interviews were conducted via phone and nine through Zoom. The duration of the interviews ranged from 15 to 42 min (mean 29 min). The majority of participating pharmacists were female (n = 12) and were practising in metropolitan areas (n = 11). Most pharmacists were based in New South Wales (n = 13), one pharmacist worked in Western Australia (n = 1), and one pharmacist worked in the Australian Capital Territory (n = 1).

**Fig. 1:**
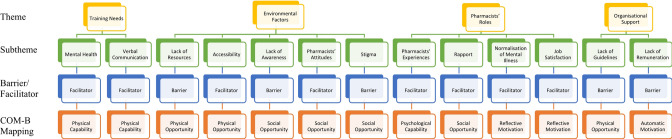
Subthemes mapped to the COM-B Model

Four key themes were identified, including *training needs, environmental factors, pharmacists’ roles*, and *organisational support*. Thirteen subthemes emerged from within these themes. These subthemes were further divided into barriers and facilitators, and mapped to the COM-B model, as highlighted in Fig. 1.

Subthemes which emerged from the data were mapped to the COM-B model by categorising whether they affected pharmacists’ capability (physical or psychological), opportunity (physical or social), or motivation (reflective or automatic) to screen older adults for depression. In this study, the subthemes impacting pharmacists’ physical and psychological capability were *mental health* and *verbal communication*, and *pharmacists’ experiences*, respectively. Subthemes linked to physical and social opportunity were *lack of resources*, *accessibility* and *lack of guidelines*, and *lack of awareness, pharmacists’ attitudes*, *stigma and rapport*, respectively. Subthemes affecting pharmacists reflective and automatic motivation were *normalisation of mental illness* and *job satisfaction*, and *lack of remuneration*, respectively.

### Physical capability

Physical capability was mapped to the theme *training needs*, and specifically to the subthemes *mental health* and *verbal communication*.

All participants highlighted the importance of appropriate mental health training. It was reported that mental health training (including MHFA) may assist in the identification of signs and symptoms of depression, thereby acting as a prompt to screen at-risk consumers, and may upskill pharmacists to respond appropriately to consumers in distress.

*“MHFA comes to be really important. If we hear something that is distressing, we know what to do and what to say.”* (P7).

Additionally, participants felt verbal communication training could contribute to their ability to approach and discuss mental health with consumers.

*“…how to engage and speak to someone - the language to use/sorts of questions to ask.”* (P6).

### Psychological capability

Psychological capability was mapped to the theme *pharmacists’ roles*, more specifically to the *pharmacists’ experiences* subtheme.

Pharmacists’ previous experience with providing mental healthcare was said to enhance their psychological capability and improve their confidence when providing such services.

*“Experience is another concern too; pharmacists may feel nervous if they haven’t had enough exposure with different patients.”* (P9).

### Physical opportunity

Physical opportunity was linked to the theme *environmental factors* and its subthemes *lack of resources* and *accessibility*, in addition to the theme *organisational support* and the subtheme *lack of guidelines*.

Most participants raised concerns about insufficient time to discuss mental health with consumers. They explained that pharmacists are constantly multitasking due to factors such as understaffing, in turn affecting their ability to deliver screening services.

*“It is always busy and fast-paced, sometimes it is very difficult to… dedicate time to provide those services.”* (P7).

Participants also noted that a lack of privacy in community pharmacies could hinder the effective uptake of depression screening services and emphasised the importance of private consultation rooms in encouraging consumers to feel comfortable and open up.

*“Once there is a private consultation room, it actually eases the worries of people who dislike discussing mental health issues.”* (P15).

Participants recognised the lack of standardised guidelines surrounding pharmacist-delivered screening as a barrier. They stated that there are no standardised guidelines for pharmacists providing LLD screening services, which may cause some hesitation about delivering these services.

*“It will be beneficial for all pharmacies to use a standardised measure… where all of them just use that guide.”* (P13).

Some participants mentioned the importance of follow-up procedures, to ensure adequate support for consumers.

*“There is no point in screening someone and saying, ‘okay you are at risk of having depression’ but without any support provided as follow up.”* (P15).

Participants viewed the accessibility of pharmacies as a facilitator to conducting pharmacist-delivered depression screening. Some participants mentioned that community pharmacies may be a preferred setting for screening, and feel less intimidating, compared to a doctor’s surgery.

*“I think community pharmacies are quite accessible, and it feels a lot less formal than a doctor’s surgery, so I think people are more likely to be themselves.”* (P15).

### Social opportunity

Social opportunity was mapped to the theme *environmental factors* and further to the subthemes *stigma, pharmacists’ attitudes*, and *lack of awareness*, as well as the theme *pharmacists’ roles* and the subtheme *rapport.*

Stigma was highlighted as a barrier by most participants. They determined that there is a significant amount of stigma associated with mental illness, and this could deter consumers from participating in screening.

*“I think there is still a large stigma about mental health and a lot of people don’t necessarily want to talk about it.”* (P15).

Some participants also highlighted the importance of appropriate terminology when approaching consumers for screening and avoiding words which may have negative connotations.

*“I would always say ‘mental wellness’ or I would say ‘you are not feeling yourself’. I feel like maybe I get a better response if I don’t say ‘depression’.”* (P1).

Participants identified a lack of awareness pertaining to the role of pharmacists in mental healthcare as a barrier, stating that most consumers do not visit pharmacies expecting to discuss their mental health.

*“I think having pharmacists approaching them is a pretty foreign thing. Initially, it is going to take them back and they may withdraw, and the opposite might happen, and it is going to be a barrier from the start.”* (P11).

It was also noted that the attitude of pharmacists may impact the acceptability of depression screening services. Some participants highlighted the importance of maintaining a non-judgemental attitude, as consumers may feel more comfortable if they felt they were speaking to a pharmacist who demonstrated a positive attitude towards mental health.

*“To provide the chance to speak up about their concerns and have someone listen to them non-judgmentally and sometimes reassuring them it’s okay to ask for help.”* (P7).

Participants stated that pharmacists may develop relationships and establish rapport with consumers. This could build trust between consumers and pharmacists and allow consumers to feel comfortable discussing their mental health with their pharmacist.

*“Building rapport with patients and then hopefully using that rapport or relationship with patients, we are able to break down those boundaries and make them more comfortable to talk about it.”* (P11).

### Reflective motivation

Reflective motivation was linked to the theme *pharmacists’ roles* and further to the subthemes *normalisation of mental illness* and *job satisfaction.*

Some participants highlighted that normalisation of mental illness could act as a motivator for pharmacists to deliver depression screening services. They stated that having open conversations about mental illness may allow pharmacists to reassure consumers and normalise mental illness.

*“Normalise the condition rather than make them feel that something’s wrong with them which is bad.”* (P6).

Additionally, some participants mentioned that professional identity and job satisfaction may motivate the implementation of depression screening services, as pharmacists are generally eager to expand their roles.

*“It is the other side of it - giving us the professional satisfaction.”* (P14).

### Automatic motivation

Automatic motivation was mapped to the theme *organisational support* and its subtheme *lack of remuneration*.

Lack of funding and monetary incentives was recognised as a barrier to the provision of pharmacist-delivered screening. Participants stated that pharmacists often provide services free of charge and may struggle to find the motivation to continue expanding pharmacy services with no incentives.

*“I feel like a monetary incentive can be helpful because pharmacists are asked to do a lot of work out of the goodness of their hearts. And I feel like people are growing very tired of it.”* (P12).

*“Funding and financial support would be one of the motivations for us to be actively involved.”* (P14).

## Discussion

### Statement of key findings

This study appears to be the first to explore community pharmacists’ acceptability of pharmacist-delivered LLD screening. Four key themes emerged which highlighted the barriers and facilitators to providing depression screening services in community pharmacies: training needs, environmental factors, pharmacists’ roles, and organisational support.

### Strengths and limitations

Both male and female pharmacists of varying ages and lengths of employment who worked in both rural and metropolitan areas in Australia were interviewed in this study, allowing the perspectives of pharmacists from different demographics to be garnered. Moreover, the COM-B model is a taxonomic framework used to develop interventions in various settings and using different research designs [28]. Use of this model allowed for the exploration of behavioural changes which may affect the effective implementation of pharmacist-delivered LLD screening services. However, only pharmacists who were employed in Australia were permitted to participate in this study. As a result, the general perspectives discussed in this study may not be representative of pharmacists working outside of Australia. Additionally, it is likely that participating pharmacists may have had a particular interest in mental health research, resulting in self-selection bias. Furthermore, this study only explored pharmacists’ views regarding pharmacy-based depression screening. To allow for triangulation, future studies may explore other stakeholders’ (e.g., consumers, GPs etc.) perspectives regarding these services.

### Interpretation

In this study, training was determined to be vital for the provision of pharmacist-delivered LLD screening, affecting pharmacists’ physical capability to deliver screening services. While training for any pharmacy-based service is important, mental health training is vital for LLD screening, due to the public stigma regarding mental illness experienced by older adults [29]. Mental health training educates pharmacists on how to use non-stigmatising language when discussing mental health with consumers [30], which has been shown to reduce stigma [31]. Mental health training may also increase pharmacists’ knowledge about mental illnesses, thereby better equipping pharmacists to identify at-risk individuals [30]. This is especially important considering the varying symptoms of depression in older and younger adults [6]. Mental health training also incorporates interactive exercises which allow pharmacists to gain practical experience regarding how to approach consumers and initiate conversations about mental health [30]. There appears to be a lack of adequate training relating to mental health included in healthcare curricula, in turn affecting the knowledge and confidence of HCPs when interacting with consumers with mental illnesses [32]. Soliman et al. recognised lack of education surrounding mental health to be the most prominent barrier relating to the role of pharmacists in depression care [33]. The importance of mental health training was also highlighted in a study by O’Reilly et al., whereby pharmacists received training before implementing depression screening services [23]. The results obtained in their study largely reflect the outcomes of this study. In particular, pharmacists acknowledged that mental health training may provide them with the skills to identify mental health-related issues and carry out post-screening referral procedures.

A lack of guidelines pertaining to depression screening services was also noted, affecting pharmacists’ physical opportunity to screen for depression. Guidelines may allow pharmacists to effectively integrate screening services in their practice and ensure appropriate post-screening procedures are followed. Nollett et al. conducted a study whereby practitioners, including optometrists, employed at a low vision rehabilitation centre were trained to screen their consumers for depression [34]. As part of their training, practitioners were provided with an overview of the relevant clinical guidelines and screening procedures. The study concluded that training on depression screening guidelines increased the number of practitioners who identified depression in consumers attending the low vision centre, thereby indicating the importance of depression screening guidelines, and signifying the need for the development of pharmacist-specific LLD screening guidelines.

Lack of resources, including privacy and time, were identified as barriers to the implementation of pharmacist-delivered LLD screening services, affecting pharmacists’ physical opportunity to provide depression screening services. Similar barriers were identified by Elkhodr et al., as lack of time and privacy were reported as significant barriers influencing the identification of women at-risk for postnatal depression [35]. However, these barriers are not specific to pharmacist-delivered depression screening services, with other studies identifying these as significant barriers affecting other pharmacy-based interventions. Karia et al. reported that the provision of pharmacy services is often hindered by lack of sufficient time due to understaffing, high workloads and frequent interruptions during the delivery of these services [36]. Hattingh et al. highlighted that privacy must be prioritised for the implementation of pharmacy services, as consumers are more likely to disclose private health information if they feel their privacy requirements are being adhered to [37]. Therefore, access to a private consultation room and the opportunity to devote more time to consumers can positively influence the delivery of pharmacy services, including depression screening services.

Stigma was also found to be a major barrier to the provision of depression screening in community pharmacies, impacting pharmacists’ social opportunity to provide LLD screening. This notion is supported in a study by Colligan et al., in which stigma was noted as a barrier which could interfere with effective depression screening [38]. Additionally, Conner et al. reported that older adults were found to experience high levels of public stigma, which prevented them from seeking mental health treatment [29]. Stigma was also reported to prevent older adults from self-reporting symptoms of mental illness [39]. As such, a non-judgemental attitude was found to be essential when performing depression screening in order to encourage open communication and trust between pharmacists and consumers [38], further supporting the findings from this study. Addressing these factors may allow for the normalisation of mental illness, acting as a reflective motivator for pharmacist-delivered LLD screening.

The lack of funding and remuneration for providing LLD screening services was seen as negatively influencing pharmacists’ automatic motivation. Dalton et al. identified that pharmacists are often not reimbursed for providing pharmacy services, listing this as a barrier to the implementation of such services [40]. The lack of reimbursement and government support was also found to affect the incorporation of pharmacists as key players in the provision of primary healthcare services [41], and to negatively impact the sustainability of pharmacist-delivered screening services [42–45]. Remuneration can therefore assist in promoting the recognition, and supporting the role, of pharmacists in mental healthcare [46]. As a result, the future development of a funding scheme may increase pharmacists’ automatic motivation to deliver depression screening services.

Rapport with consumers present pharmacists with the social opportunity to formulate relationships with consumers. Tarn et al. noted that older adults often confide in pharmacists regarding medication-related problems before contacting their GPs, and trust pharmacists to provide information about their medications and current treatment plans [47]. Furthermore, Mey et al. found that trust between the consumer and pharmacist was achieved through the establishment of rapport and forming a relationship over time, and this ultimately led consumers to feel more comfortable discussing personal health-related issues [14]. This is noteworthy, as Mickus et al. found in their study exploring the general public’s preferences for mental health services providers, that 10% of older adult respondents did not know where to seek mental healthcare [48]. Therefore, the relationship between pharmacists and consumers may enable the effective delivery of these services.

Increased job satisfaction was identified as a reflective motivator for pharmacist-delivered LLD screening. Job satisfaction has been shown to greatly impact HCPs’ quality of, and commitment to, work [49]. Participants revealed that engaging in the delivery of screening services heightened their sense of professional identity. Similar notions were reported in a study by Lowres et al. who reported that pharmacists who provided an atrial fibrillation screening service experienced increased job satisfaction through the more thorough interactions that accompany screening and by learning new skills to effectively implement the screening service [19]. Likewise, Elliott et al. revealed that pharmacists who delivered osteoporosis screening programs reported an increased sense of professional satisfaction [50]. In this way, we can infer that pharmacists’ engagement in the provision of screening services may enhance their job satisfaction, thereby motivating them to deliver screening services.

### Further research

This study utilised the COM-B model to understand the core behavioural practices affecting the implementation of pharmacist-delivered LLD screening services. Future studies may further link such findings to the Behaviour Change Wheel to assess the feasibility of these services. Furthermore, future studies may explore consumers’ perspectives regarding pharmacist-delivered LLD screening services, as well as the development of standardised guidelines and funding schemes to provide support for pharmacists delivering LLD screening services.

## Conclusion

This study provided insight into community pharmacists’ acceptability of pharmacist-delivered LLD screening. Application of the COM-B model allowed for the identification of behavioural changes which may affect the provision of LLD screening services in community pharmacies. As this is a novel area of research, this study provided important insight demonstrating that while there are factors affecting pharmacist-delivered screening in general, such as lack of resources and remuneration, there are special considerations necessary specifically for LLD screening, such as mental health training focusing on depression in older adults and the public stigma regarding mental illness experienced by older adults. Addressing the barriers and facilitators identified in this study may help to facilitate the implementation of LLD screening services in community pharmacies and may ultimately increase the early identification and treatment of LLD.

### Electronic supplementary material

Below is the link to the electronic supplementary material.

Supplementary file 1 (DOCX 17 kb)
