# Supplementary material for: Community pharmacists’ acceptability of pharmacist-delivered depression screening for older adults: a qualitative study
Source: Int J Clin Pharm. 2023 Apr 20;45(5):1144–52. doi: 10.1007/s11096-023-01581-1 (PMC10600303; doi:10.1007/s11096-023-01581-1)
Supplement: Supplementary file 1 — (DOCX 17 kb) [file 11096_2023_1581_MOESM1_ESM.docx]

**Supplementary material 1 – Interview guide**

1. Can you tell me briefly about your role/experience in community pharmacy?

2. Does your pharmacy have a private consultation room/area to discuss sensitive issues around mental illness? *(Further explore their opinions on providing privacy in depression screening services, can you tell me more about your opinion on protecting privacy during conversations around mental health?)*

3. As a pharmacist, how could you go about trying to reduce the stigma and discrimination that can exist with mental illnesses in the community?

4. In your opinion, how do you as a community pharmacist provide services in mental health care?

5. Do you feel that community pharmacists can play a key role in providing mental health services, why or why not?

6. In your opinion, how should a pharmacist encourage consumers to disclose their mental health problems (i.e. depression)?

7. What kind of role do you think pharmacists should have in the early intervention of depression for older Australians (≥65 years)?

8. Do you commonly interact with consumers in this age group that might be at risk of depression?

If so, how confident are you to consult your patients about the risk of depression in a community pharmacy setting? *(Ask them to further explore issues that may affect their confidence; Ask how did their concern arises? How satisfied they were in handling such situations).*

9. Have you ever used a depression screening tool in the past? If YES - what’s your opinion on that experience?

10. Could you elaborate more regarding the advantages, and disadvantages of the screening tool(s) you’ve used in the past, including things that can be improved in that screening tool(s)?

11. How did your consumer respond to that depression screening service?

12. What guidelines, or standard operating procedure does your community pharmacy adopt for supporting pharmacists to identify the early signs of depression? *(If they’ve answered no, ask what sort of guidelines/ support do you expect your workplace to provide you in assisting with a depression screening service?)*

13. What do you think are the most important training elements for community pharmacists who wish to be involved in depression screening?

14. What do you think some of the barriers to a depression screening service might be?

a) Why do you think these barriers or challenges arose?

b) How do you think these barriers could be overcome in the future?
